# Supplementary material for: Quantifying the drivers and predictability of seasonal changes in African fire
Source: Nat Commun. 2020 Jun 9;11:2893. doi: 10.1038/s41467-020-16692-w (PMC7283213; doi:10.1038/s41467-020-16692-w)
Supplement: Supplementary file 1 — Supplementary Information [file 41467_2020_16692_MOESM1_ESM.pdf]

Supplementary Information for:

Quantifying the drivers and predictability of seasonal changes in African fire

Yu and Mao et al. (2020)

Supplementary Table 1 Summary of the observational, reanalysis, and remote sensing datasets applied in SGEFA analysis and MLTs-based prediction. Select references on each previously identified predictor are listed.

**Variable**	**Dataset**	**Period**	**Spatial Resolution**
Response Variable			
Fire-related carbon emission	Global Fire Emissions Database (GFED)[1]	1997-2016	0.25˚x0.25˚
Burned area fraction	Global Fire Emissions Database (GFED)[1]	1997-2016	0.25˚x0.25˚
Currently Identified Environmental Predictors (SGEFA Forcings)			
Sea-surface temperature (SST)	Hadley Centre Sea Ice and Sea Surface Temperature data set (HadISST)[2]	1870-2018	1˚x1˚
Leaf area index (LAI)	Global Inventory Monitoring and Modeling System (GIMMS) 3rd generation LAI (LAI3g)[3]	1982-2015	0.0833˚x0.0833˚
	Long-term Global Mapping LAI (GLOBMAP LAI)[4]	1982-2018	0.0833˚x0.0833˚
	National Oceanic and Atmospheric Administration (NOAA) Climate Data Record (CDR) of AVHRR LAI[5]	1982-2018	0.05˚x0.05˚
Surface-layer soil moisture	Global Land Evaporation Amsterdam Model (GLEAM) v3.3a[6]	1980-2018	0.25˚x0.25˚
	European Centre for Medium-Range Weather Forecast (ECMWF) Interim Reanalysis (ERA-Interim)[7]	1979-2018	0.75˚x0.75˚
Previously Identified Climatic Predictors			
Monthly-mean 2-m air temperature[8,9]	University of Delaware (UDEL) Terrestrial precipitation v5.01[10]	1900-2017	0.5˚x0.5˚
	University of East Anglia Climatic Research Unit (CRU) high resolution gridded data TS4.03[11]	1901-2018	0.5˚x0.5˚
Precipitation[8,9]	UDEL v5.01[12]	1900-2017	0.5˚x0.5˚
	CRU TS4.03[11]	1901-2018	0.5˚x0.5˚
	Global Precipitation Climatology Project (GPCP) v2.3[13]	1979-2017	2.5˚x2.5˚
	Global Precipitation Climatology Centre (GPCC) v2018[14]	1891-2016	0.5˚x0.5˚
Monthly mean of daily minimum and maximum 2-m air temperature, diurnal temperature range, wet day frequency, potential evapotranspiration, and vapor pressure[8,9]	CRU TS4.03[11]	1901-2018	0.5˚x0.5˚

2-m wind speed[9]	National Aeronautics and Space Administration (NASA) Modern-Era Retrospective Analysis for Research and Applications (MERRA) v2[15]	1980-2018	0.625˚x0.5˚
Monthly flash rate[16]	Lightning Imaging Sensor (LIS) on the Tropical Rainfall Measurement Mission (TRMM)[17]	1997-2015	0.5˚x0.5˚
Previously Identified Socio-economical Predictors			
Annual land-use statics, including fractions of forested primary land, managed pasture, rangeland, urban land, and cropland, and their changes compared with last year[8]	Land use harmonization (LUH2 v2h)[18]	850-2100	0.25˚x0.25˚
Population density[8]	History Database of the Global Environment (HYDE v3.2)[19]	10,000 BCE to 2017 CE	0.0833˚x0.0833˚

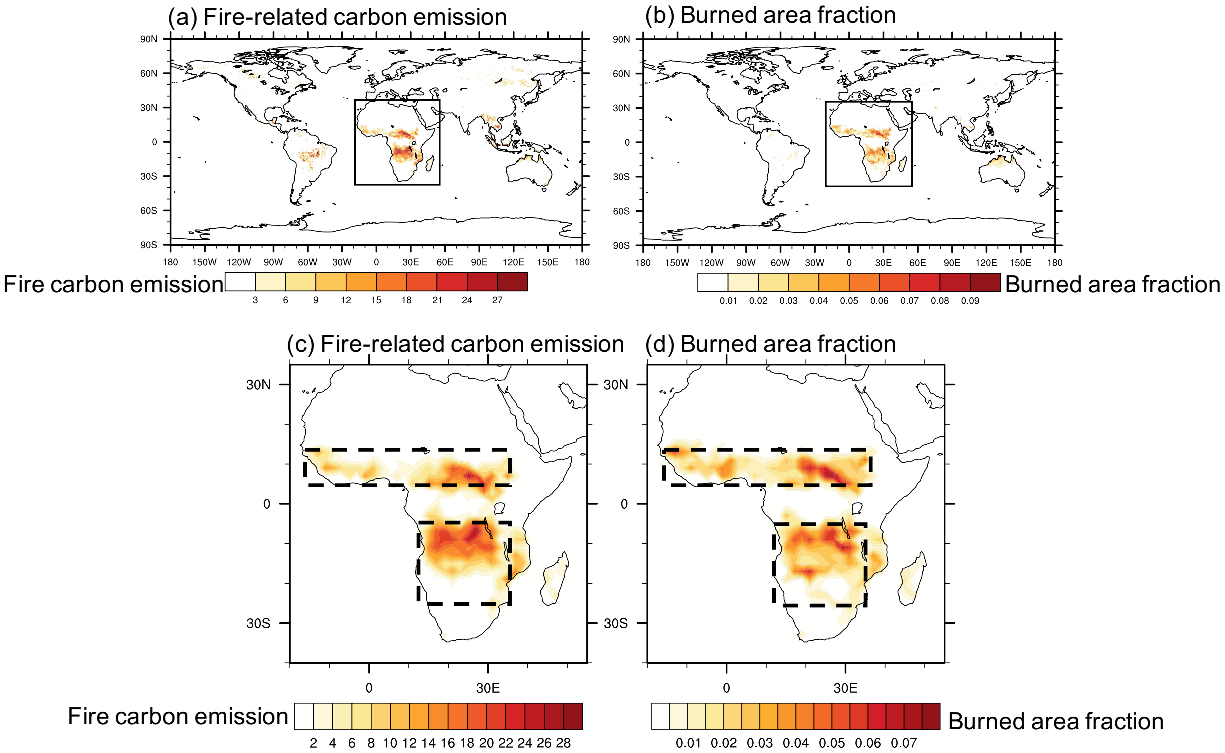

Supplementary Figure 1: Annual mean global and African fire activity. Annual mean (a, c) fire-related carbon emission (kg m^{-2} mon^{-1}) and (b, d) burned area fraction in (a, b) the globe and (c, d) Africa during 1997-2016.

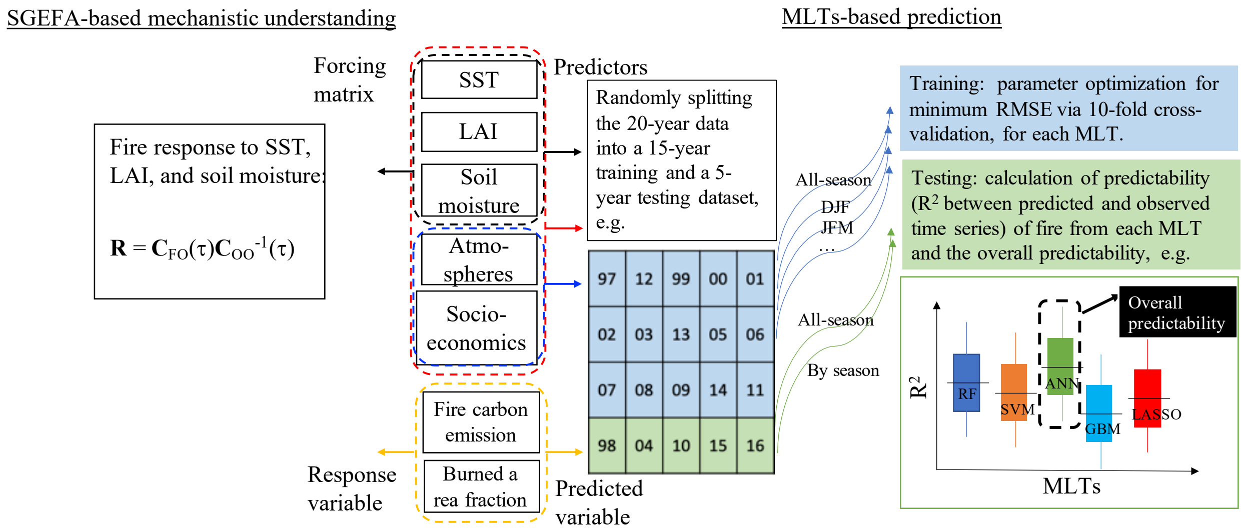

Supplementary Figure 2: Schematic of the current analytical framework.

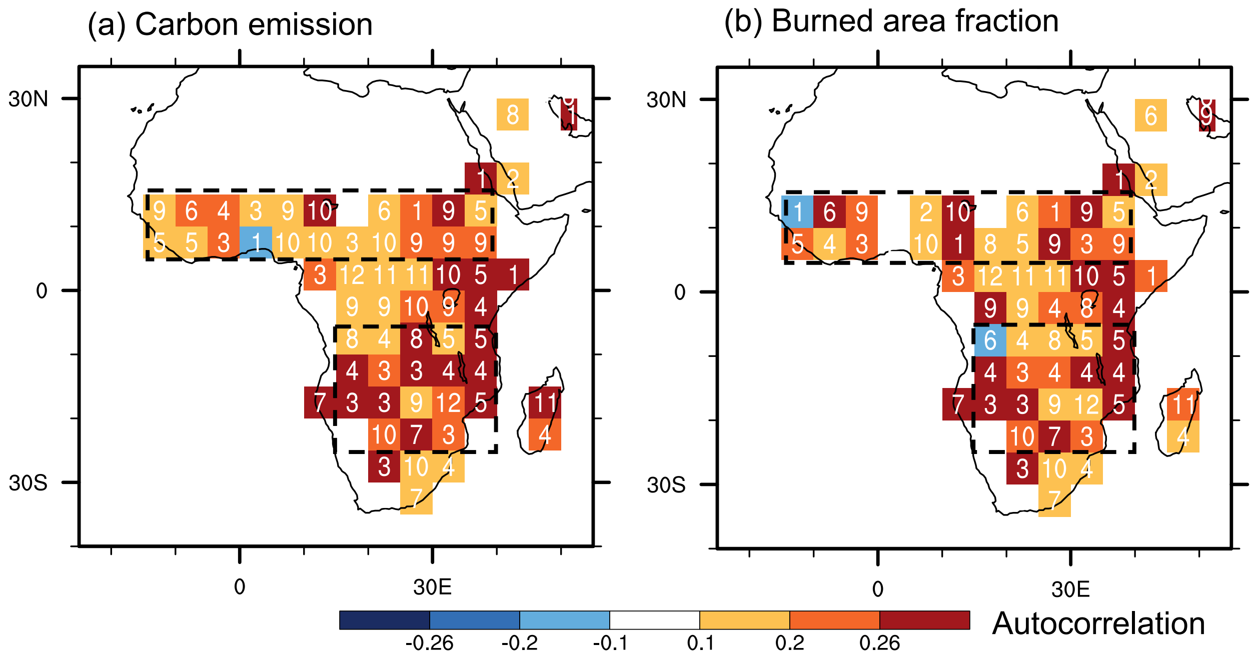

Supplementary Figure 3: Summary of one-month autocorrelation in African fire. The analyzed variables include (a) Fire carbon emission and (b) burned area fraction by season. Color stands for the maximum autocorrelation magnitude. Number indicates the season of maximum autocorrelation magnitude. Autocorrelations with absolute magnitude larger than 0.26 are statistically significant (n=60, p<0.05) according to the two-tailed Student's t-test. Regions with insignificant autocorrelations in both fire carbon emission and burned area exhibit higher credibility of the current Stepwise Generalized Equilibrium Feedback Assessment (SGEFA) results. The boxes indicate the geographic location of the northern and southern African ecoregions in the present study.

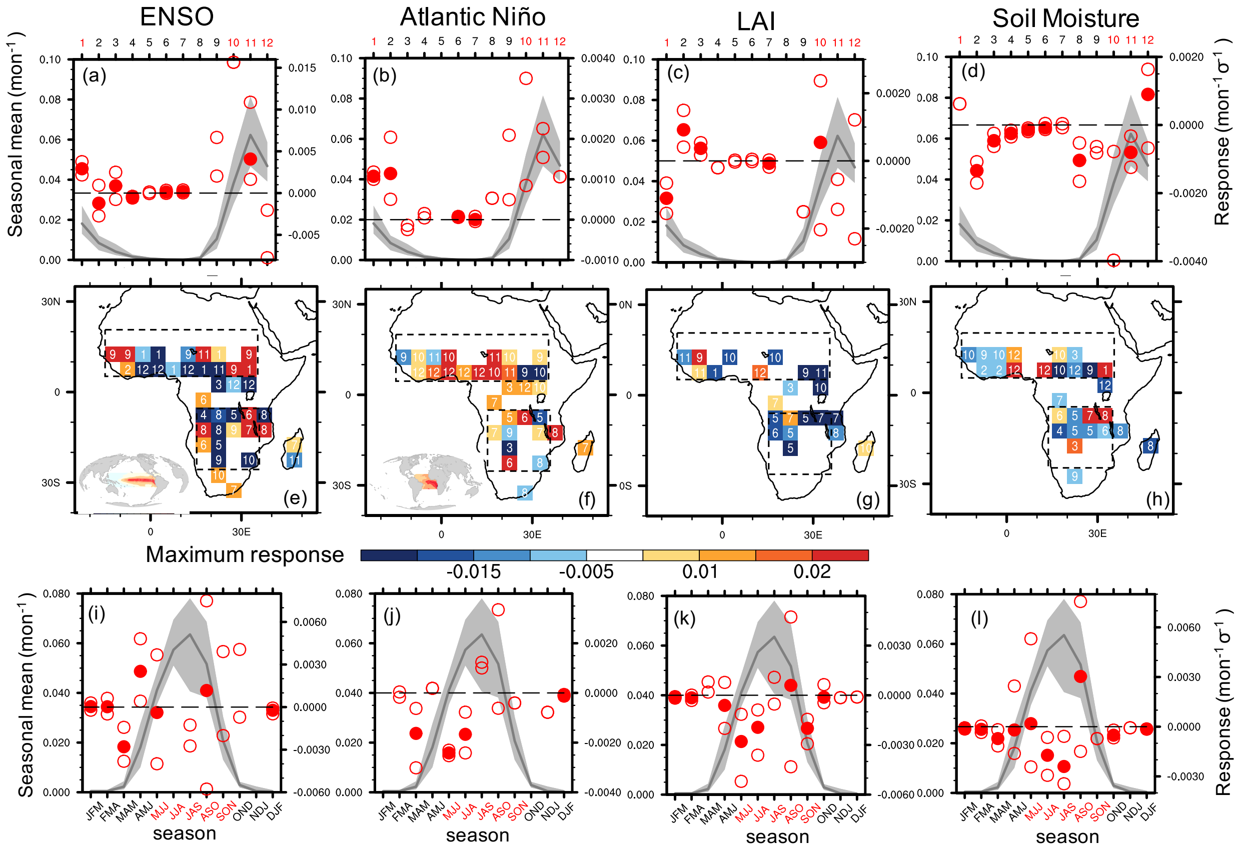

Supplementary Figure 4: African fire response to key environmental drivers. Response of African burned area fraction to the most important environmental forcings is assessed by the Stepwise Generalized Equilibrium Feedback Assessment (SGEFA). These forcings include (a, e, i) El Niño–Southern Oscillation (ENSO), (b, f, j) Atlantic Niño mode, (c, g, k) leaf area index (LAI), and (d, h, l) soil moisture. Seasonal cycle of the (a–d) northern and (i–l) southern African regional average climatology, with line and shading representing mean and interannual standard deviation, referring to the left *Y*-axis (unit: kgC m^{-2} $month^{-1}$), and response to the corresponding forcing, with the filled and open circles indicate the multi-dataset average and 10th and 90th percentiles, referring to the right *Y*-axis (unit: $month^{-1}$ $\sigma_{forcing}^{-1}$). A missing filled circle in (a-d) and (i-l) indicates insignificant multi-dataset average response to the specific forcing. (e–h) Spatial distribution of the season (number representing each three-month season, e.g. 1 for January-March) and sign and magnitude (color) of the maximum absolute response to the corresponding forcing. The spatial patterns of the ENSO and Atlantic Niño forcing are demonstrated in the inserted global map in (e) and (f), respectively. In these inlets, postive and negative anomalies in sea-surface temperature are represented by red and blue colors respectively. The boxes in (e–h) indicate the geographic location of the currently assessed northern and southern African ecoregions. Only statistically significant responses ($p<0.1$) are shown here. In i-l, labels on the x-axis stand for three-month seasons, e.g., JFM for January, February, and March. The fire-active season is highlighted with red labels on the x-axis.

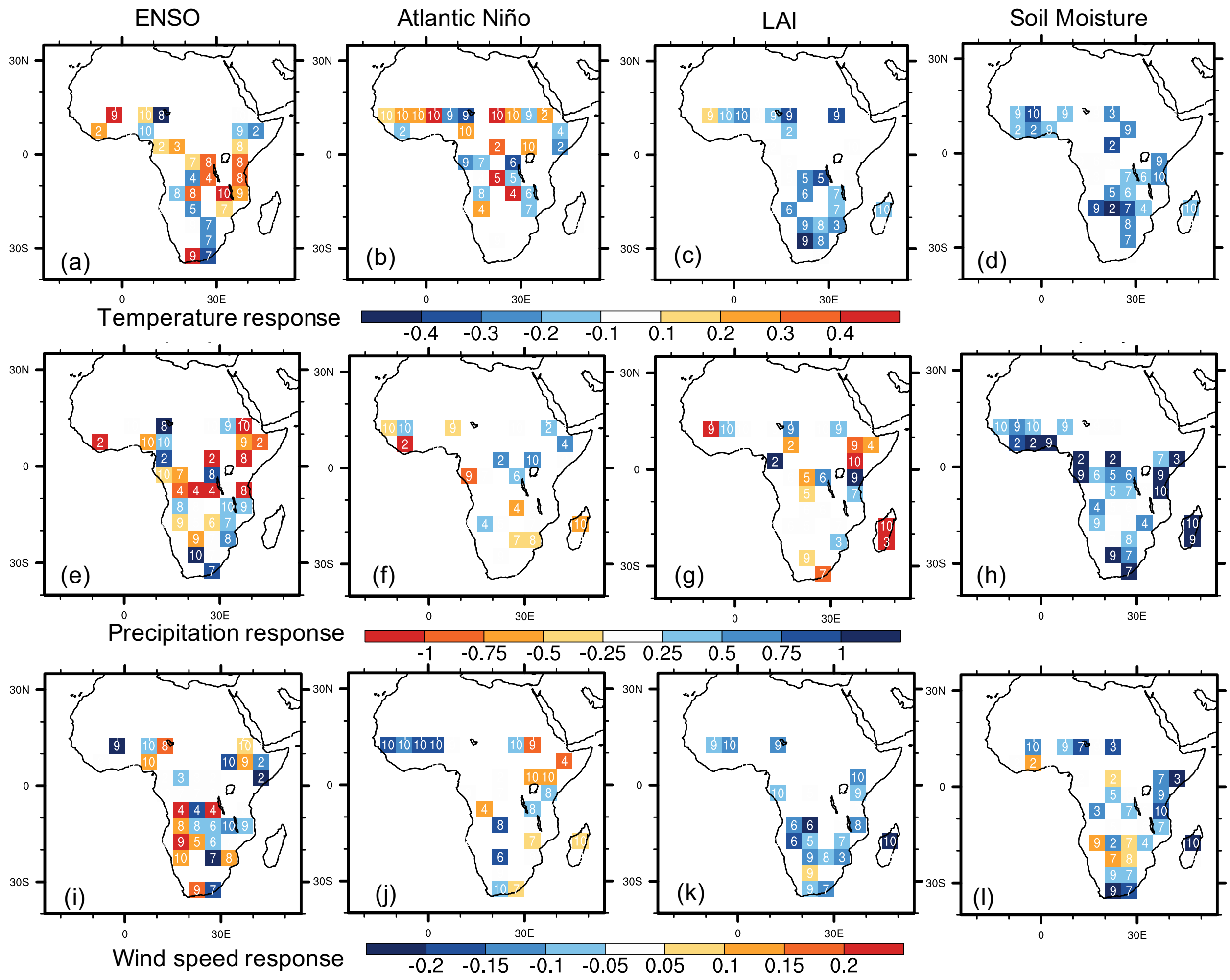

Supplementary Figure 5: Response of regional climate to key environmental forcings. Responses in (a-d) 2-m air temperature (˚C $\sigma_{forcing}^{-1}$), (e-h) recipitation (mm day^{-1} $\sigma_{forcing}^{-1}$), and (i-l) 2-m wind speed (m s$^{-1}\sigma_{forcing}^{-1}$) to select environmental forcings are assessed by the Stepwise Generalized Equilibrium Feedback Assessment (SGEFA), and shown here in the season with corresponding maximum response in fire carbon emission. These forcings include (a, e, i) El Niño–Southern Oscillation (ENSO), (b, f, j) Atlantic Niño mode, (c, g, k) LAI, and (d, h, l) soil moisture. Only statistically significant ($p < 0.1$) responses are shown here. Numbers represent each three-month season, e.g. 1 for January-March, when the absolute response in fire carbon emission maximizes. Colors represent the sign and magnitude of the climatic response to the corresponding forcing in the corresponding season.

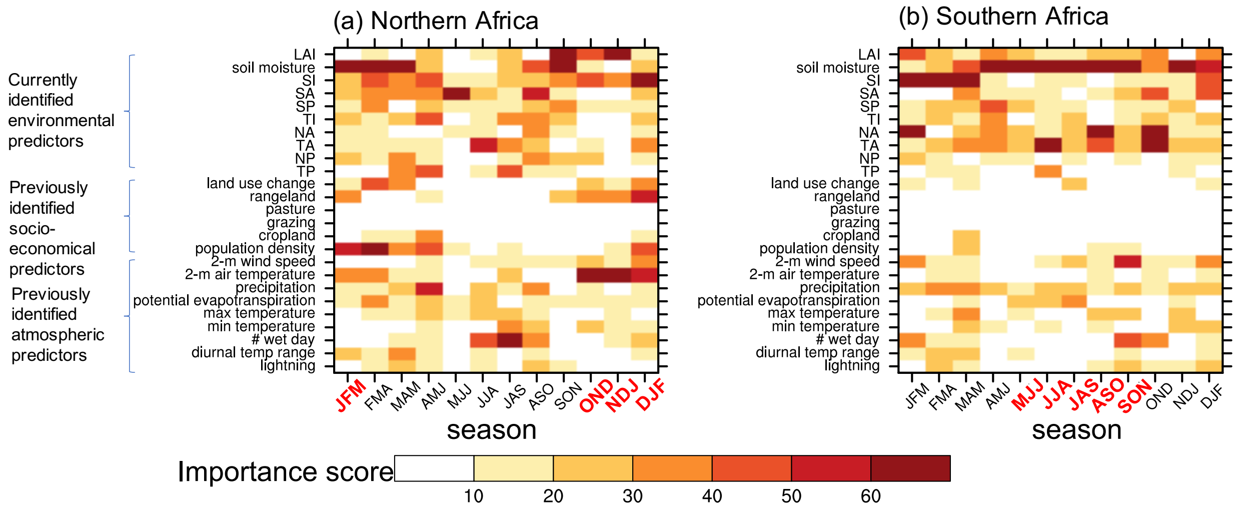

Supplementary Figure 6: Importance of fire carbon emission predictors by season. The importance of predictors is a standard output of all Machine Leaning Techiniques (MLTs). Although the calculation of importance scores varies substantially by MLT, all of the importance scores qualitatively reflect relative importance of each predictor. Here the importance score for each predictor in each season is calculated as the ensemble-average importance score reported by the best MLT. For the environmental and climatic predictors, the maximum importance scores across different antecedent time windows are shown. The fire-active season is highlighted on the x-axis labels.

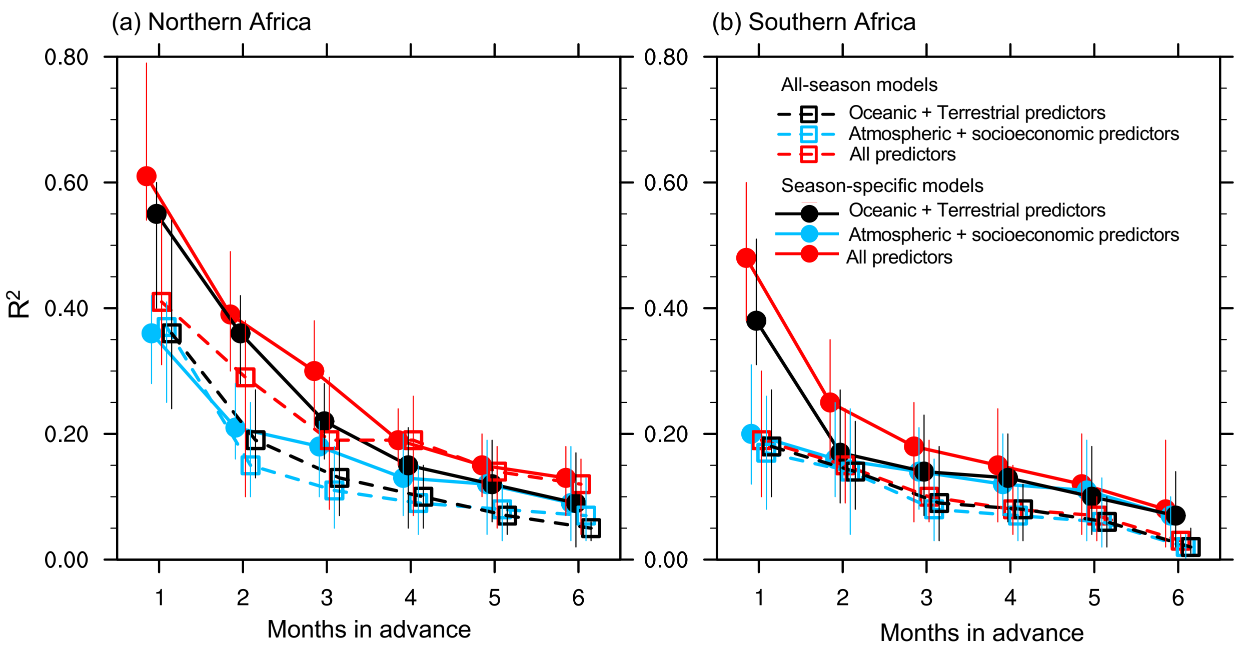

Supplementary Figure 7: **Predictability of African fire by leading time.** The predictability of African burned area fraction anomalies is estimated using multiple machine learning techniques (MLTs) as a function of lead time. The predictability is represented by the squared correlation coefficient (R^2) between the predicted and observed monthly anomalies (n = 60) of the regional average burned area fractions across the (a) northern and (b) southern African ecoregions. The assessed sets of predictors include previously identified atmospheric and socioeconomic predictors (blue), currently identified oceanic and terrestrial predictors (black), and the combination of all these predictors (red). The assessed models include season-specific models (filled circles), in which the MLTs are built and applied by season,and all-season models (open squares). The circles and squares indicate the mean R^2 across the 100 ensemble members of the best MLT (Methods), and the vertical lines indicate the range of 10th and 90th percentiles of the 100 ensemble members.

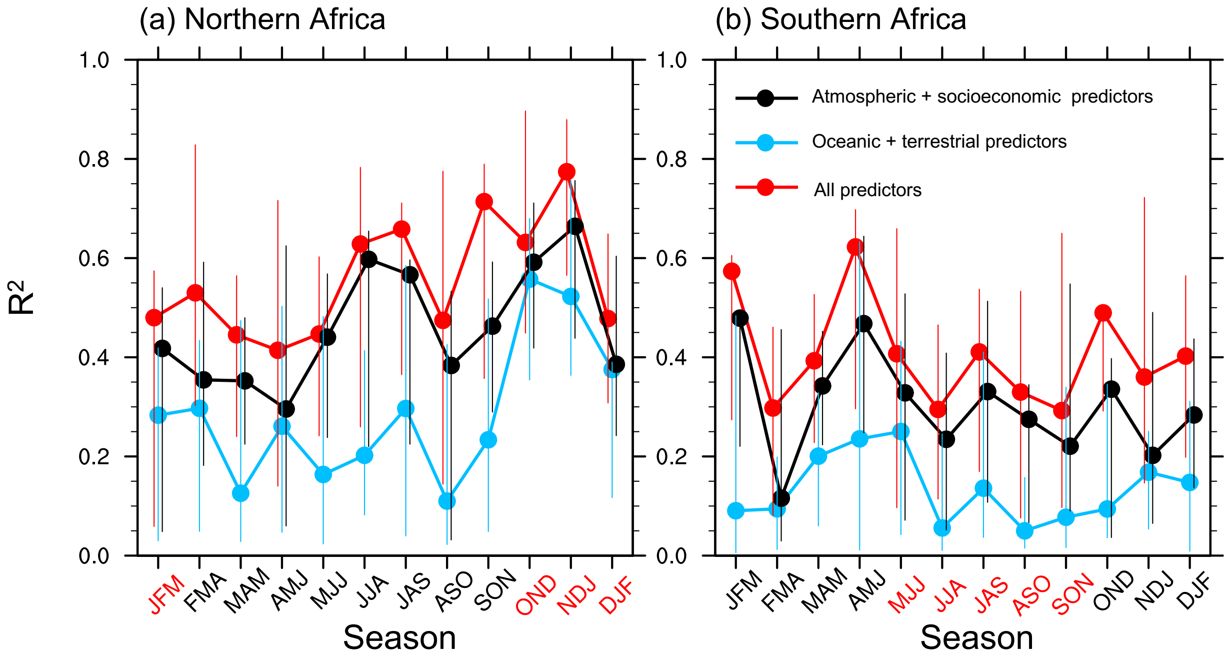

Supplementary Figure 8: **Predictability of African fire anomalies by season.** The predictability of regional average burned area fraction anomalies in each season is estimated using multiple machine learning techniques (MLTs). The predictability is represented by the squared correlation coefficient (R^2) between the predicted and the observed monthly anomalies (n = 15, Methods) of the regional average burned area fraction across the (a) northern and (b) southern African ecoregions. The assessed sets of predictors include previously identified atmospheric and socioeconomic predictors (blue), currently identified oceanic and terrestrial predictors (black), and the combination of all these predictors (red). Season-specific prediction models are assessed here at one month in advance. The filled circles represent the mean R^2 across the 100 ensemble members of the best MLT, and the vertical indicate the range of 10th and 90th percentiles of the 100 ensemble members. The fire-active season is highlighted with red labels on the x-axis.

Supplementary References

1. Van Der Werf, G. R. *et al.* Global fire emissions and the contribution of deforestation, savanna, forest, agricultural, and peat fires (1997-2009). *Atmos. Chem. Phys.* **10**, 11707–11735 (2010).
2. Rayner, N. A. Global analyses of sea surface temperature, sea ice, and night marine air temperature since the late nineteenth century. *J. Geophys. Res.* **108**, (2003).
3. Zhu, Z. *et al.* Global data sets of vegetation leaf area index (LAI) 3g and Fraction of Photosynthetically Active Radiation (FPAR) 3g derived from Global Inventory Modeling and Mapping Studies (GIMMS) Normalized Difference Vegetation Index (NDVI3g) for the period 1981 to. *Remote Sens.* **5**, 927–948 (2013).
4. Liu, Y., Liu, R. & Chen, J. M. Retrospective retrieval of long-term consistent global leaf area index (1981-2011) from combined AVHRR and MODIS data. *J. Geophys. Res. G Biogeosciences* **117**, (2012).
5. Vermote, E. & NOAA CDR Program. NOAA Climate Data Record (CDR) of AVHRR Leaf Area Index (LAI) and Fraction of Absorbed Photosynthetically Active Radiation (FAPAR), Version 5. doi:https://doi.org/10.7289/V5TT4P69
6. Martens, B. *et al.* GLEAM v3: Satellite-based land evaporation and root-zone soil moisture. *Geosci. Model Dev.* **10**, 1903–1925 (2017).
7. Dee, D. P. *et al.* The ERA-Interim reanalysis: Configuration and performance of the data assimilation system. *Q. J. R. Meteorol. Soc.* **137**, 553–597 (2011).
8. Forkel, M. *et al.* A data-driven approach to identify controls on global fire activity from satellite and climate observations (SOFIA V1). *Geosci. Model Dev.* **10**, 4443–4476 (2017).
9. Di Giuseppe, F. *et al.* The potential predictability of fire danger provided by numerical weather prediction. *J. Appl. Meteorol. Climatol.* **55**, 2469–2491 (2016).
10. Matsuura, K. & Willmott, C. J. Terrestrial Air Temperature: 1900-2017 Gridded Monthly Time Series. (2018). Available at: http://climate.geog.udel.edu/~climate/html_pages/Global2017/README.GlobalTsT2017.html.
11. Harris, I., Jones, P. D., Osborn, T. J. & Lister, D. H. Updated high-resolution grids of monthly climatic observations - the CRU TS3.10 Dataset. *Int. J. Climatol.* **34**, 623–642 (2014).
12. Matsuura, K. & Willmott, C. J. Terrestrial Precipitation: 1900-2017 Gridded Monthly Time Series. (2018). Available at: http://climate.geog.udel.edu/~climate/html_pages/Global2017/README.GlobalTsP2017.html.
13. Huffman, G. J. *et al.* The Global Precipitation Climatology Project (GPCP) Combined Precipitation Dataset. *Bull. Am. Meteorol. Soc.* **78**, 5–20 (1997).
14. Schneider, U. *et al.* GPCC's new land surface precipitation climatology based on quality-controlled in situ data and its role in quantifying the global water cycle. *Theor. Appl. Climatol.* **115**, 15–40 (2014).
15. Rienecker, M. M. *et al.* MERRA: NASA's Modern-Era Retrospective Analysis for Research and Applications. *J. Clim.* **24**, 3624–3648 (2011).
16. Taylor, S. W., Woolford, D. G., Dean, C. B. & Martell, D. L. Wildfire prediction to inform fire management: Statistical science challenges. *Stat. Sci.* **28**, 586–615 (2013).
17. Cecil, D. J., Buechler, D. E. & Blakeslee, R. J. Gridded lightning climatology from TRMM-LIS and OTD: Dataset description. *Atmos. Res.* **135–136**, 404–414 (2014).
18. Hurtt, G. C. *et al.* Harmonization of land-use scenarios for the period 1500-2100: 600 years of global gridded annual land-use transitions, wood harvest, and resulting secondary lands. *Clim. Change* **109**, 117–161 (2011).
19. Goldewijk, K. K., Beusen, A., Doelman, J. & Stehfest, E. Anthropogenic land use estimates for the Holocene - HYDE 3.2. *Earth Syst. Sci. Data* **9**, 927–953 (2017).
